# Microstructure and tribological behaviour of CoCrCuFeTi high entropy alloy reinforced SS304 through friction stir processing

**DOI:** 10.1038/s41598-024-54267-7

**Published:** 2024-02-13

**Authors:** N. Radhika, S. Aravind Krishna, Animesh Kumar Basak, Adeolu Adesoji Adediran

**Affiliations:** 1 Department of Mechanical Engineering, Amrita School of Engineering, Amrita Vishwa Vidyapeetham, Coimbatore, India; 2 Adelaide Microscopy, The University of Adelaide, Adelaide, SA Australia; 3 Department of Mechanical Engineering, Landmark University, P.M.B. 1001, Omu-Aran, Kwara State Nigeria; 4 Department of Mechanical Engineering Science, University of Johannesburg, Johannesburg, South Africa

**Keywords:** Friction stir processing, High entropy alloys, Tribology, Wear resistance, Annealing, Engineering, Materials science

## Abstract

Surface modification by suitable technique aids in improving the characteristics of material to resist severe wear in demanding environments and challenging applications. The present study aims to analyse the tribological performance of Stainless Steel (SS304) reinforced with CoCrCuFeTi High Entropy Alloy (HEA) through friction stir processing and compares the results with annealed specimens. The CoCrCuFeTi HEA was ball milled and revealed irregular fragment particles with Body Centred Cubic (BCC) phase. The processed samples exhibited excellent refinement in grains with uniform HEA reinforcement distribution. The grains were observed to be in nano level post-annealing promoting exceptional microhardness. The pin-on-disc wear test was conducted by varying load (10-40N), sliding velocity (0.5–3.5 m/s) and sliding distance (500–2000 m) and the respective worn surface was analysed. The processed sample with HEA after annealing offered 29.8%, 57.4% and 58.49% improved wear resistance at the minimum level of load, sliding velocity and sliding distance than the processed base samples. The worn morphology revealed delamination, abrasion, adhesion and oxide layer formation to be the predominant wear mechanisms.

## Introduction

Neoteric domain of material science research explores expanding the functionality of the resources to maximize the efficiency and performance by various processing techniques that alleviate the defects. Steels used in hydraulic machinery, turbine blades in hydraulic power plants and fluid handling systems endure wear and serve as the main failure mode^1,2^. Erudite studies adduce property enhancement of the base materials can be achieved through processing techniques. This drives the need to expand the processing methods on base metals^3,4^. One common approach is the coating of the surface which provides a protective layer over the surface from wear and corrosion. But, under extreme conditions for a protracted period, the odds of the coating getting peeled off stand high^5,6^. The strategy to resolve this issue turned the research towards surface modification routes where the external layer of the material itself gets modified at the micro level. Friction Stir Processing (FSP) is a promising surface modification route favouring refinement of the surface layer at the microstructure stage. FSP refines the surface through the heat evolved from friction with no additional heat input or release of toxic fumes in the process^7,8^. This attribute of FSP made it considered to be a green process and green fabrication technique^9^. The heat generated in the FSP also greatly influence the strength and properties^10^. Pan et al. carried out FSP on stainless steel and obtained grain refinement at a sub-micron level with a maximum microhardness of 779HV. The refinement of grains and the dissolution of particles improved the corrosion resistance of the material as well^11^. Xie et al. carried out FSP on duplex stainless steel and observed ultra refinement in grains due to dynamic recrystallisation with a large amount of high angle grain boundary. The yield strength of the processed steel was comparable with the base metal due to the formation of microvoids^12^. Further exploring, FSP followed by a heat treatment process has resulted in homogeneous and defect-free microstructure aiding in the improvement of properties. Annealing is a competent heat treatment process to redefine the grains and eliminate any micro stress^13,14^. Xue et al. analysed the mechanical characteristics of carbon steel after FSP followed by annealing at 500 °C and 600 °C for 2 h. The microstructural characterisation revealed equiaxed grains with homogeneous distribution and the ultrafine grains were retained after the annealing process with increased carbide particles. It also revealed an apparent improvement in microhardness and tensile strength than the base metal^15^.

Howbeit, FSP on steel is limited to surface modification with no filler to a large extant and very few studies with typical ceramic filler reinforcement. The stimulus to develop novel material established a unique alloy termed HEA. HEA is formed by five or more elements blended in equiatomic or near equiatomic ratio till a single phase is obtained. The odds to form intermetallic with such an intricate alloy system inferred from phase diagrams are negated by experimental trials where the higher entropy of mixing advances single-phase solid solution^16–18^. HEAs can also possess complex phase structures and micro or nano precipitates depending on the processing techniques. These nanoprecipitates can improve the strength of the alloy^19^. HEAs are processed by various techniques which includes ball milling, vacuum melting, atomisation, and so on^20^. Wei et al. synthesised AlCoCrFeNi HEA by ball milling and attained Body Centred Cubic (BCC) dominant phase after milling for 20 h and the amorphous phase after 84 h of milling. The powder particle size was decreased till 20 h and increased after 20 h of milling resulting in irregular flakes^21^. CrFeNiMnCu HEA prepared by induction melting and resulted in triplex microstructure strengthened by nano precipitates leading to a hierarchical structure with improved mechanical properties^22^. Studies have reported an improvement in tribological properties after FSP of steels that support the utilisation of the technique in the mentioned applications. Tinubu et al. processed stainless steel by FSP and observed refined grains with an average size of 2–3 µm which subsequently improved microhardness by 34%. The improved microhardness contributed to better wear performance through microabrasion in the sample^23^. Aldajah et al. equipped FSP on high carbon steel to improve wear strength by modifying the surface. The wear rate was significantly decreased after the FSP on dry conditions and even more decreased under lubrication^24^.

The amassed literature infers that property is enhanced through FSP. The influence of HEA on the steel material through FSP and wear related studies of steel through FSP are limited. This provides a scope to study the influence of HEA as reinforcement through FSP. This study marks the first attempt to analyse the tribological behaviour of stainless steel with CoCrCuFeTi HEA reinforced through FSP and annealing. Thus, the present study discusses the microstructural characterisation and tribological analysis of steel processed by FSP with CoCrCuFeTi HEA as reinforcement. The tribological behaviour of the base sample is compared against the FSP sample before and after annealing.

## Materials and methods

### Materials and FSP

**Figure 1:**
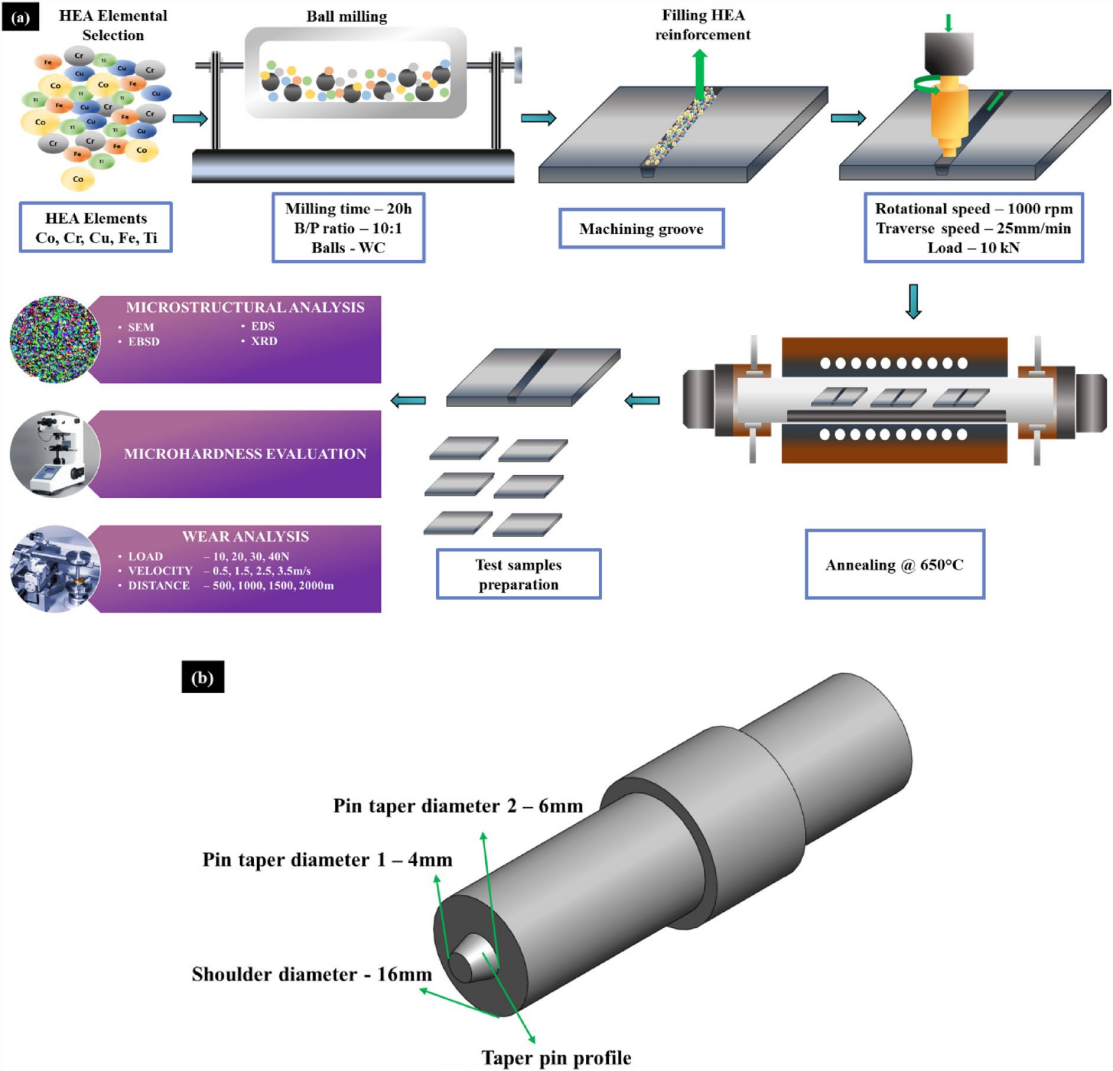
(**a**) Significant processes involved in the devised methodology (**b**) FSP tool.

A HEA comprising elements such as Co, Fe, Cr, Cu and Ti was synthesised by ball milling carried out for 20 h. The elements Ti and Cu increase mechanical strength through grain strengthen^25,26^. The addition of Fe, Co and Cu improves plasticity and Cr with Ti promotes wear resistance by providing scratch resistance^27,28^. The elements were chosen concerning their intrinsic properties that contribute to better wear performance. Stainless steel grade 304 (SS304) was chosen as the base metal to conduct the FSP process based on the application mentioned. The elemental distribution in SS304 is listed in Table 1. SS304 plate of 100 × 100 mm with 6 mm thickness was machined with a mechanical groove of trapezoid profile for a depth of 3 mm to add the HEA filler by wire cut Electric Discharge Machining (EDM). The proposed methodology of the current study is depicted in Fig. 1a. The FSP process was carried out with a tungsten carbide tool rotating at 1000 rpm with a traverse speed of 25 mm/min and a load of 10 kN on the LMW Kodi-40 machine from Coimbatore, Tamil Nadu, India. The processing parameters were optimised and considered from previous studies that infers better properties can be achieved at high rotating speed and low traverse speed with optimum load^29,30^. The pin diameter and shoulder diameter of the tool were 4 mm and 16 mm with a tapered cylinder profile (Fig. 1b). The FSP was done on both base metal and HEA-filled plates. The processed base metal and HEA-filled metal were annealed at 650 °C for 2 h. The annealing conditions were optimised from previous findings^15,31^. The different samples used in this study are designated with codes as tabulated in Table 2.

**Table 1:** SS304 chemical composition.

Elements	Cr	C	Mo	Si	Cu	Mn	Ni	V	Fe
Wt. %	17.9	0.05	0.005	0.5	0.05	2	8.3	0.1	Bal

**Table 2:** Test sample codes used in this study.

	Sample	Code
Before annealing	Processed base metal	PBM
	Processed sample with HEA	PHM
After annealing	Processed base metal	ABM
	Processed sample with HEA	AHM

### Performance analysis

The microstructural characterisation was executed by Scanning Electron Microscope (SEM), Energy Dispersive x-ray Spectroscopy (EDS) and X-Ray Diffraction (XRD) analysis. The morphology was analysed by Gemini 300 SEM and the elemental verification by the EDS detector integrated with SEM apparatus. XRD was done on the Empyrean-made XRD equipment. The grain morphology of the samples was analysed by Orientation Imaging Microscopy (OIM) and Electron Back Scatter Diffraction (EBSD) analysis using Quanta 3D FEG equipment with a step count of 0.5 µm. The microhardness of the processed samples was evaluated on the cross sectioned sample from the stir zone to the base metal in the Vickers scale using the Mitutoyo MVK H11 microhardness tester according to ASTM 384 standard. The microhardness was measured from the indentation by applying 100 g load for 15 s. The wear test was performed on a pin-on-disc wear tester from Ducom Instruments (model—TR—20LE—PHM 200) as per ASTM G-99 standard under normal temperature. The counter material used was EN32 steel with 65HRC hardness. The chosen wear parameters were load, sliding velocity and sliding distance. The applied load was altered from 10 to 40 N, the sliding velocity was altered from 0.5 m/s to 3.5 m/s and the sliding distance was altered from 500 to 2000 m. The load was adjusted by adding weights in the load cell. The sliding velocity and sliding distance were controlled on the wear monitor & tester and the tests were conducted. The tribological behaviour was analysed through specific wear rate (SWR) and coefficient of friction (COF). The weight of the wear test samples is measured before and after the wear test to calculate the SWR. The COF is calculated from the ratio of frictional force to the normal force. The worn surfaces of the test samples are characterised by SEM to study the morphology of the worn surface.

## Results and discussion

### Microstructural characterisation

**Figure 2:**
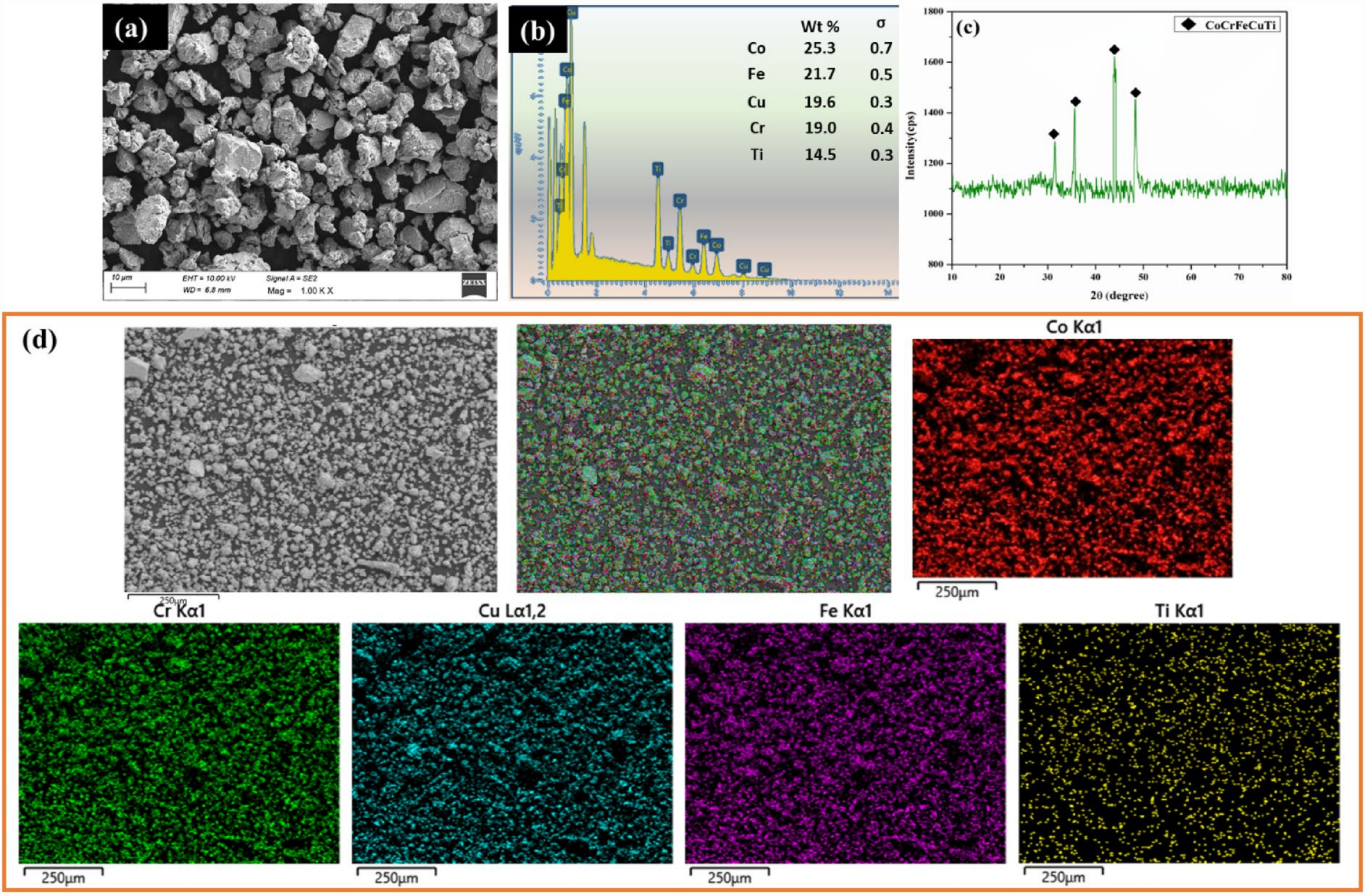
(**a**) SEM image (**b**) EDS mapping (**c**) XRD image (**d**) elemental mapping at high magnification of CoCrCuFeTi HEA particles.

**Figure 3:**
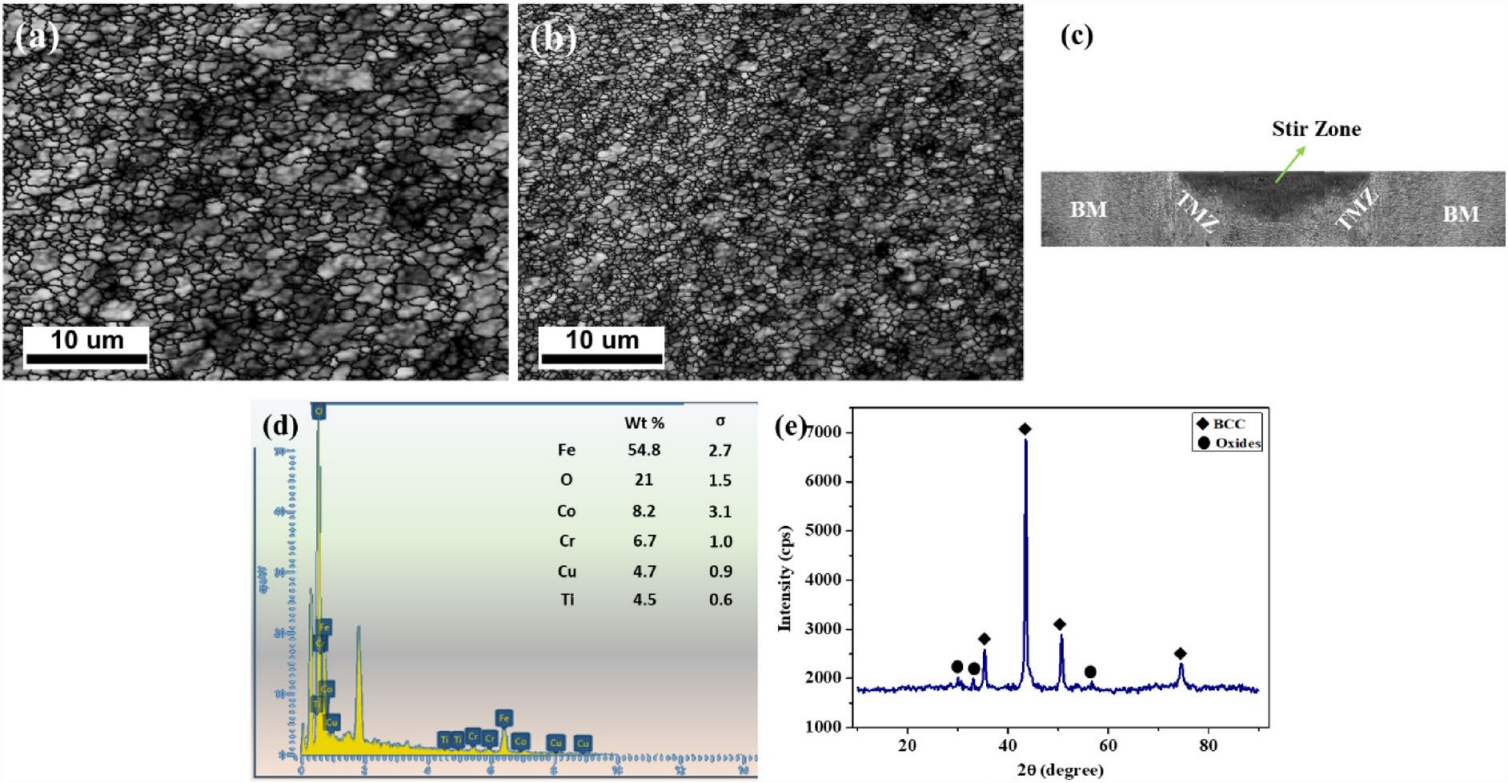
(**a**) OIM image of PHM (**b**) OIM image of AHM (**c**) cross sectional view (**d**) EDS mapping (**e**) XRD peaks of AHM.

The SEM characterisation of the ball-milled CoCrCuFeTi HEA powder is depicted in Fig. 2a. The irregular fragments of the HEA are likely from the ball milling process and the average particle size is 17 µm. It is observed that the synthesised HEA powder is dense in nature. The EDS mapping of the powder confirms the presence of each of the elements in a defined ratio and verifies the absence of any impurities from the process (Fig. 2b). The XRD image results that the HEA exhibit the BCC phase and validates the formation of HEA (Fig. 2c). The distribution of HEA elements in the particles are shown in Fig. 2d. The crystallite size of HEA particles was calculated using Williamson-Hall equation as shown in Eq. (1),

1 $${\beta }_{h/k}{\text{cos}}\theta = \frac{K \lambda }{D}+4\varepsilon \,{\text{sin}}\theta$$

where $\beta$ is full width at half maximum of each phase, $\lambda$ is the wave length, $\theta$ is the diffraction angle, K is the shape coefficient, $\varepsilon$ is the micro strain and D is the crystallite size. The crystal size of the CoCrCuFeTi HEA is estimated as 94 nm. The microstructure of the PHM is shown in Fig. 3a. The microstructure of the AHM is shown in Fig. 3b. The OIM image shows the reinforced HEA particles in dark structures and proves that the HEA particles are distributed uniformly in the processed zone. It is evident that annealing process further enhanced the grain structure. The refinement in grains is also observed which is due to the serious plastic deformation and dynamic recrystallisation. The discontinuous dynamic recrystallisation mechanism was observed to be the recrystallisation mechanism since the stacking fault energy of SS304 is low making it hard to form continuous dynamic recrystallisation. The annealing process has contributed to the refinement of grains through static recovery and the evolution of strain-free subgrains. The different zones after FSP namely stir zone, thermo mechanical zone (TMZ) and base metal is shown in macrostructural cross section of AHM (Fig. 3c). The EDS mapping of the AHM confirms the presence of all the elements and the complete blend of the HEA reinforcements into the substrate (Fig. 3d). The BCC phase along with few minor oxide phases were revealed from the XRD analysis (Fig. 3e).

### EBSD analysis

**Figure 4:**
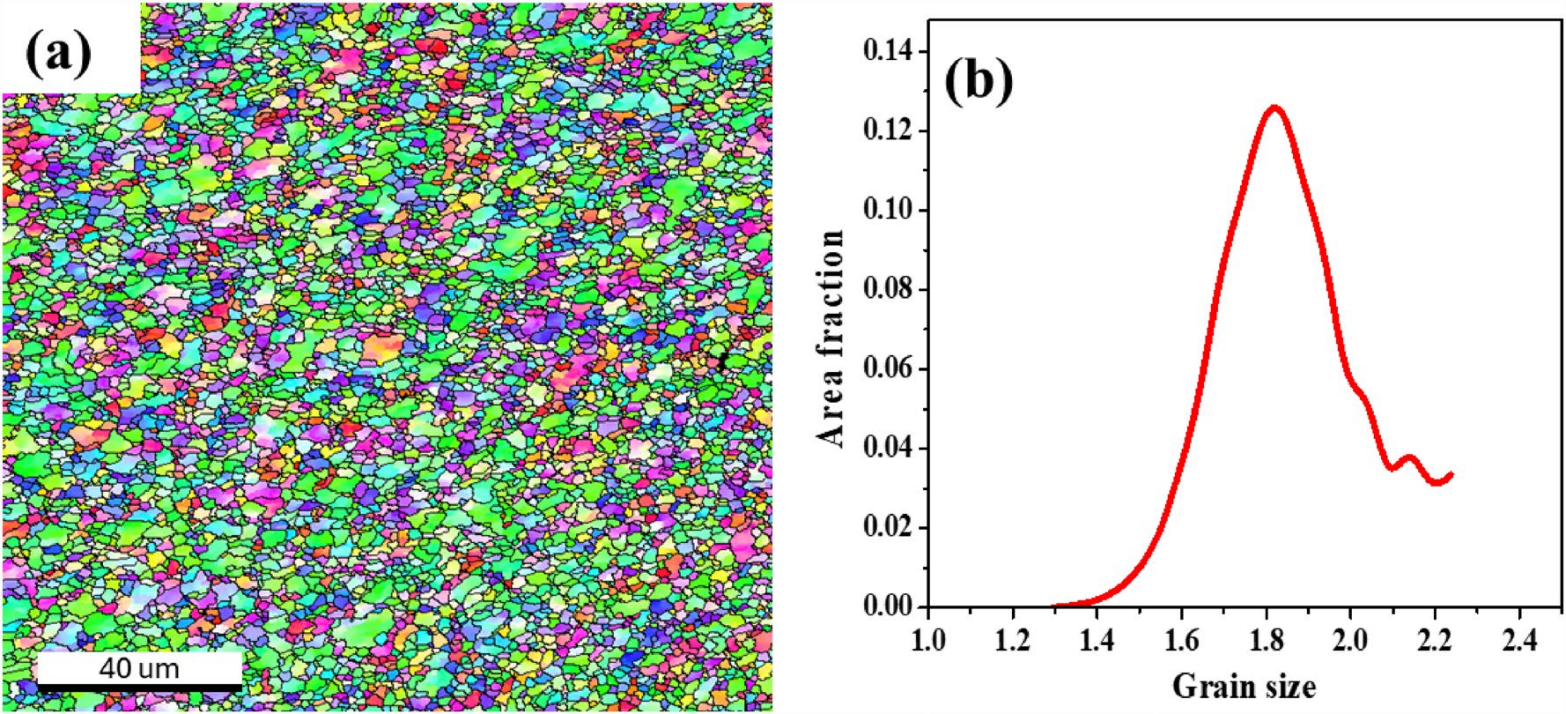
(**a**) EBSD mapping (**b**) grain size distribution of PHM.

**Figure 5:**
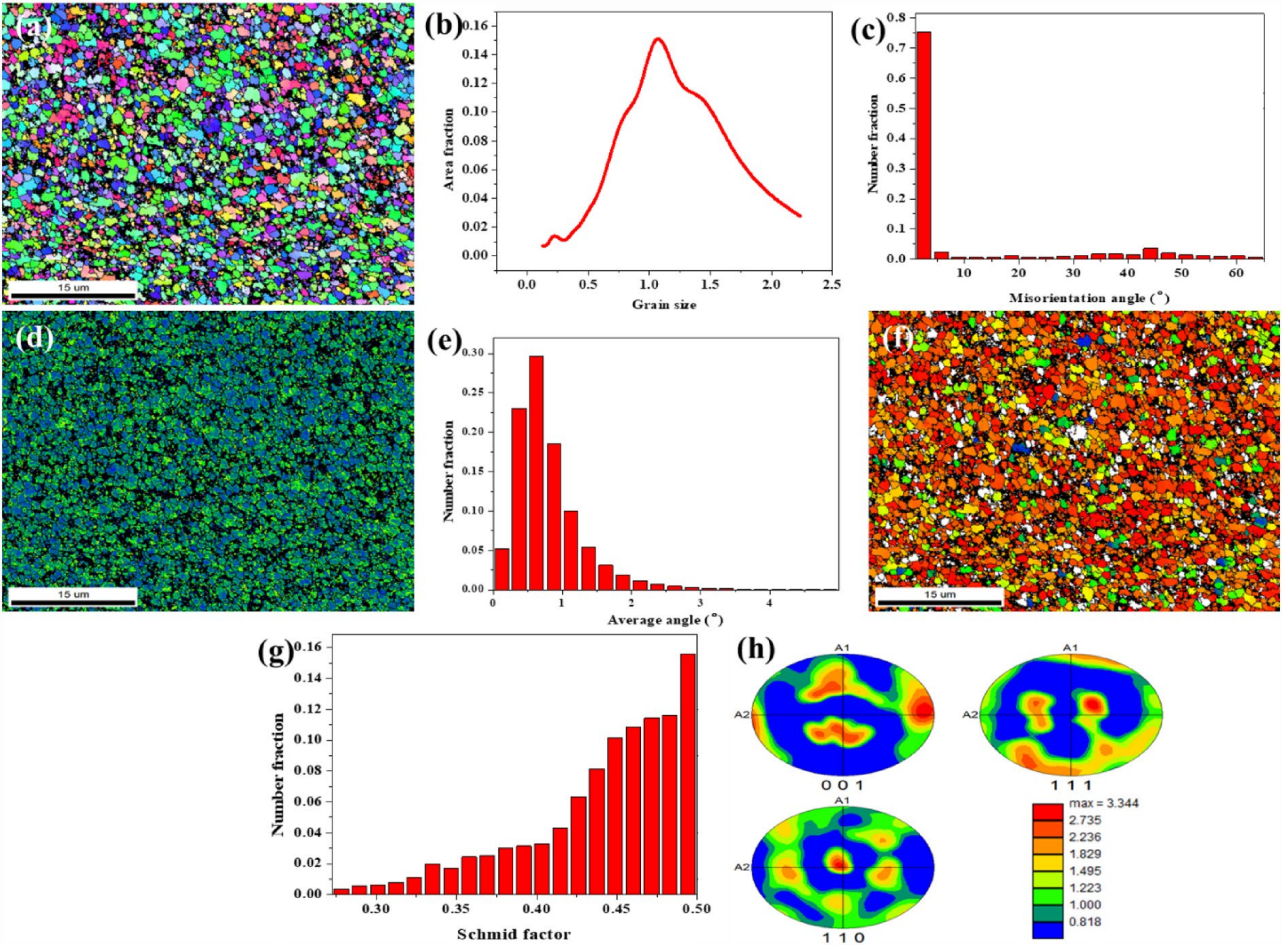
(**a**) IPF image (**b**) distribution chart of grain size (c) distribution chart of misorientation angle (**d**) KAM mapping figure (**e**) KAM distribution (**f**) Schmid factor mapping (**g**) Schmid factor distribution (**h**) pole figures of AHM.

The EBSD analysis was conducted on a 10 × 10 mm sample machined from the centre of the processed zone and the outcomes are illustrated. The EBSD analysis of PHM is shown in Fig. 4. The EBSD mapping reveals the refined grains after FSP (Fig. 4a). The grains are observed as fine equiaxed grain under micron and sub-micron level. The grain size distribution shows grain size ranges from 15 µm to 22 µm with an average grain size of 16 µm (Fig. 4b). The EBSD analysis of the AHM is shown in Fig. 5. The EBSD image of the AHM evince the characteristics of the refined grain structure. The Inverse Pole Figure (IPF) mapping presents the discrete grains toned by their crystallographic directions against the rolling direction with respect to the triangular direction code as shown in Fig. 5a. The grain structures can be observed as equiaxed grains and few coarse structures. Dynamic recrystallisation aids in the formation of fine and uniform grain structures in the stir zone of the specimen. The grain size distribution graph shown in Fig. 5b affirms that the grains size ranges from 0.12 µm to 1.34 µm with an average grain size of 0.6 µm. The inclusion of HEA particles originated the dislocation by the formation of subgrains progressing the grain refinement. The addition of HEA filler as secondary particles into the substrate convinces the recrystallisation by driving the formation of nucleation sites for new grains through particle stimulated nucleation mechanism^32^. The dislocations due to the presence of HEA also favoured the nucleation mechanism. Relevant study also reported recrystallisation by particle stimulated nucleation and barrier to grain growth by HEA enhances the grain refinement^33^. During annealing, the reinforced HEA particles hold back the migration of grain boundaries through the Zener pinning effect and similar outcomes are reported in related studies^34–36^. This leads to the breakdown of grain boundaries and ends up in forming low angle grain boundaries. The grains structures exhibit major low angle grain boundary with 75.37% grains and the density of grain boundary angles is depicted in Fig. 5c. Kernel Average Misorientation (KAM) evaluates the level of recrystallisation and plastic deformation in the FSP process. KAM mapping also presents the connect between microstrain and lattice distortion within a crystal structure after plastic deformation^37,38^. KAM mapping resulted lower values citing low strain and misorientation supporting the characteristics improvement (Fig. 5d,e). The lower average KAM values hint lower density of dislocations, which supports the way that the subsequent microstructures sustained almost completed recrystallisation and aligns with the similarly reported studies^39,40^. The Schmid factor mapping and the respective Schmid factor distribution are depicted in Fig. 5f and g. The high value of the Schmid factor is indicated by the red colour. It is observed from the distribution that the average Schmid factor values are around 0.44. The < 001 > , < 111 > and < 110 > pole figures of the processed surface after annealing are shown in Fig. 5h. The maximum texture strength is beheld to be 3.344 which is relatively lower. Lower texture strength indicates the formation of discontinuous dynamic recrystallisation. Hence, the weak texture intensity validates the occurrence of discontinuous dynamic recrystallisation and similar results are observed in relevant studies^41,42^.

### Microhardness analysis

**Figure 6:**
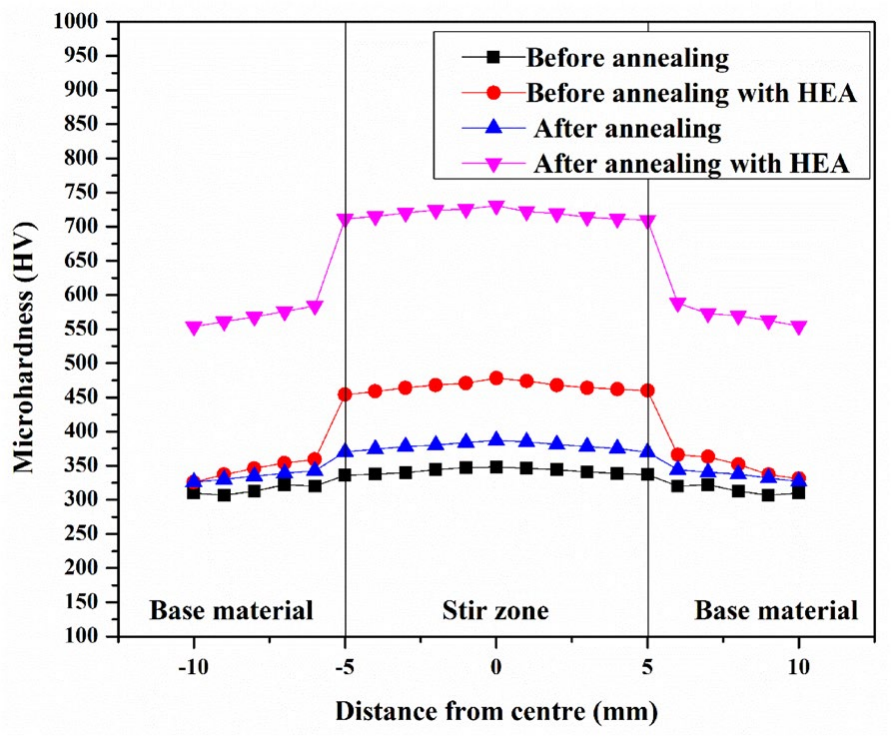
Microhardness analysis of the processed samples.

The Vickers microhardness of the AHM was measured and compared against the other processed sample. The microhardness of the processed samples is presented in Fig. 6. The microhardness improvement upon the addition of CoCrCuFeTi HEA is apparent from the graph and is credited to the grain refinement alongside the HEA addition. The AHM exhibits 52% and 88% improved microhardness than the PHM and PBM. The HEA particles induce dislocation and hinder grain growth. The annealed sample processed with HEA shows even better microhardness. This is due to the grain refinement achieved during the annealing where the heat energy relieves the stress (Fig. 5a). The increased dislocation density through high plastic deformation enhances the microhardness^43^. The reduced grain size improves the microhardness which adheres to the Hall–Petch relation^44^. The grain refinements can be verified and validated from Figs. 3 and 5. The dislocation-strengthening mechanism involved in the process also aids in the improvement of microhardness. The synergistic effect of the ultrafine refined grains by the FSP process and the stress relieved microstructures after annealing are credited to the improvement in microhardness. A similar study reflects the microhardness improvement upon the inclusion of HEA reinforcement credited to the ultra-refinement of grains and the effect of reinforcement^45^. The HEA reinforced, quench hardening effect and grain refinement are credited to the improved microhardness.

### Wear performance

The wear performance of the PBM, PHM, ABM and AHM was analysed by calculating the SWR and the COF values. The attained values are plotted as a graph and the trend observed is analysed to construe the wear behaviour in each case and the wear mechanism related to the samples. The reinforced HEA and its high entropy effect propels the wear resistance of the processed samples.

#### Effect of load on the wear rate

**Figure 7:**
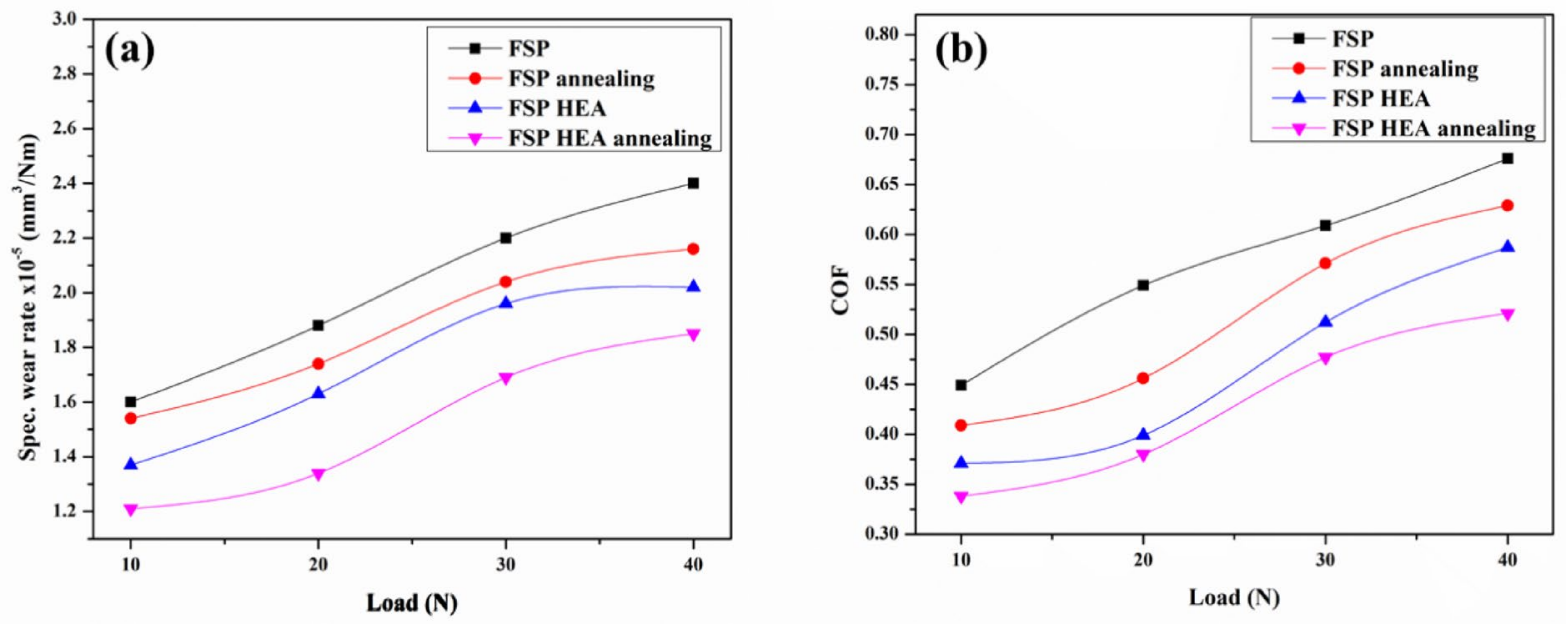
Effect of applied load on (**a**) SWR (**b**) COF.

The influence of the applied load on the SWR is analysed by varying the load from 10 to 40N with constant sliding velocity and sliding distance at 1.5 m/s and 1000 m respectively. The trend for SWRs against different loads is depicted in Fig. 7a. It is apparent that the SWR raises with raise in load applied. The AHM evinces 11% and 24.3% decreased SWR than the PHM and PBM. The PBM has the highest SWR due to the prolonged contact and friction created. The ABM has slightly better wear resistance due to the influence of annealing. The annealing process improves the grain structure that attributes to the decreased SWR than the base metal. The PHM and AHM showed decreased SWR than the base samples. The connection between the hardness and the wear resistance is deeply rooted where the harder material will more often show greater resistance to wear compared to softer material. The infused HEA act as the hard reinforcement reducing the contact area between the pin and the counterpart. The surface roughness of the counterpart also impacts the SWR. At higher applied loads, the rough surface of the counterpart induces severe wear on the pin surface^46^. The linear trend in the SWR indicates that the plastic delamination and abrasion involved to be mechanism of wear^47^. The increased load induce temperature at the contact area and generates plastic delamination on the pin surface. Though the local temperature at the surface of contact is high due to the great load applied and friction, the Ti content in the reinforced HEA offers high hot hardness and the SWR is relatively lower than the base sample^48^. The attained BCC structure in both HEA and the processed sample provides improved wear resistance which is noticed in a related study^49^. The BCC structure offers resistance to delamination and plastic deformation enabling oxide layers to endure abrasion^50^. As mild contact is made at low load conditions, the annealed samples with HEA undergo trivial loss of material due to the ultra-grain refinement and increased microhardness, whereas, the stress in the pin surface builds up and the deformation resistance drops with a load increment. The COF against load is shown in Fig. 7b. Comparing the trends, the AHM exhibits lower COF followed by the PHM. The COF trend is attributed to the plastic delamination that occurred at the pin contact surface. Since frictional force is lower and higher at low and high load conditions, the COF values at low and high load conditions are seen to lower and higher. The AHM showed better COF values than the other samples due to the better microhardness attained after annealing and the reinforced HEA particles offering better wear resistance.

#### Effect of sliding velocity on the wear rate

**Figure 8:**
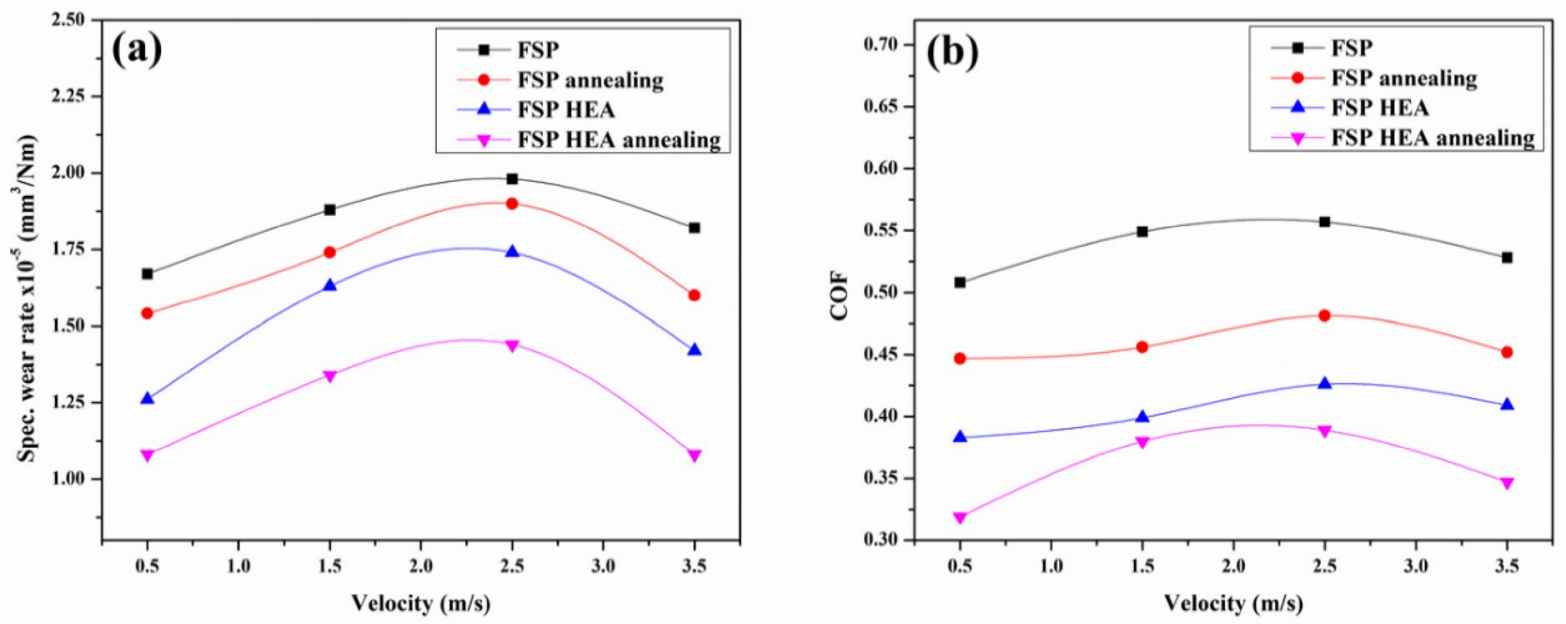
Effect of sliding velocity on (**a**) SWR (**b**) COF.

The effect of sliding velocity on the SWR of the samples is analysed by varying the sliding velocity from 0.5 m/s to 3.5 m/s by keeping the load and sliding distance at 20N and 1000 m constant. The obtained SWR is plotted against the varying velocity as shown in Fig. 8a. The SWR of the AHM is 20.5% and 35.3% lower than PHM and PBM. The SWR for the PBM and ABM increases with an increase in sliding velocity and drops after 2.5 m/s. This is because of the increased contact between the pin and the counterpart causing critical loss of material. Similarly, in PHM and AHM, the SWR raises initially to 2.5 m/s and then drops. This is attributed to the adhesive wear mechanism involved. Adhesive wear happens when two surfaces slide against one another, making a transfer of material from one surface to the other, bringing about the development of a third body layer. The third body layer comprises material from the two surfaces, and it can go about as an abrasive and create additional wear. This wear mechanism is exacerbated at higher sliding velocities, as the expanded frictional force between the surfaces improves the probability of material exchange and third body layer formation^51,52^. The reinforced HEA induces dislocation when the surface temperature is raised due to friction thereby reducing the residual stress built in. This in turn reduces the SWR of the samples. The Cu and Ti present in the reinforced HEA tends to form oxides rapidly and advance the third body layer formation which eventually increases the wear resistance^53^. At intermittent sliding velocity, the local heat at the contact surface raised and debilitated the bond between the reinforcement and the matrix leading to easy material removal. Also, the addition of HEA particles and the attained refinement in microstructure promote the heat transfer at high speed on the pin surface providing resistance to wear. Figure 8b shows the COF values against sliding velocity. The COF gradually increases initially and follows a downward trend after 2.5 m/s. At lower sliding velocity, the COF is decreased due to the lower frictional force from the minimum contact of the pin to the counter plate surface. Also, the scarcity of energy to advance surface deformation from the low frictional force at lower sliding velocity reduced the COF at the initial condition. Increasing the sliding velocity, the adhered surface from the counter plate acts as a defensive layer to wear. This stands as the reason for the reduction in COF values after initial conditions. The third body layer formed during the prolonged contact lowers the SWR after a specific point thereby reducing the COF.

#### Effect of sliding distance on the wear rate

**Figure 9:**
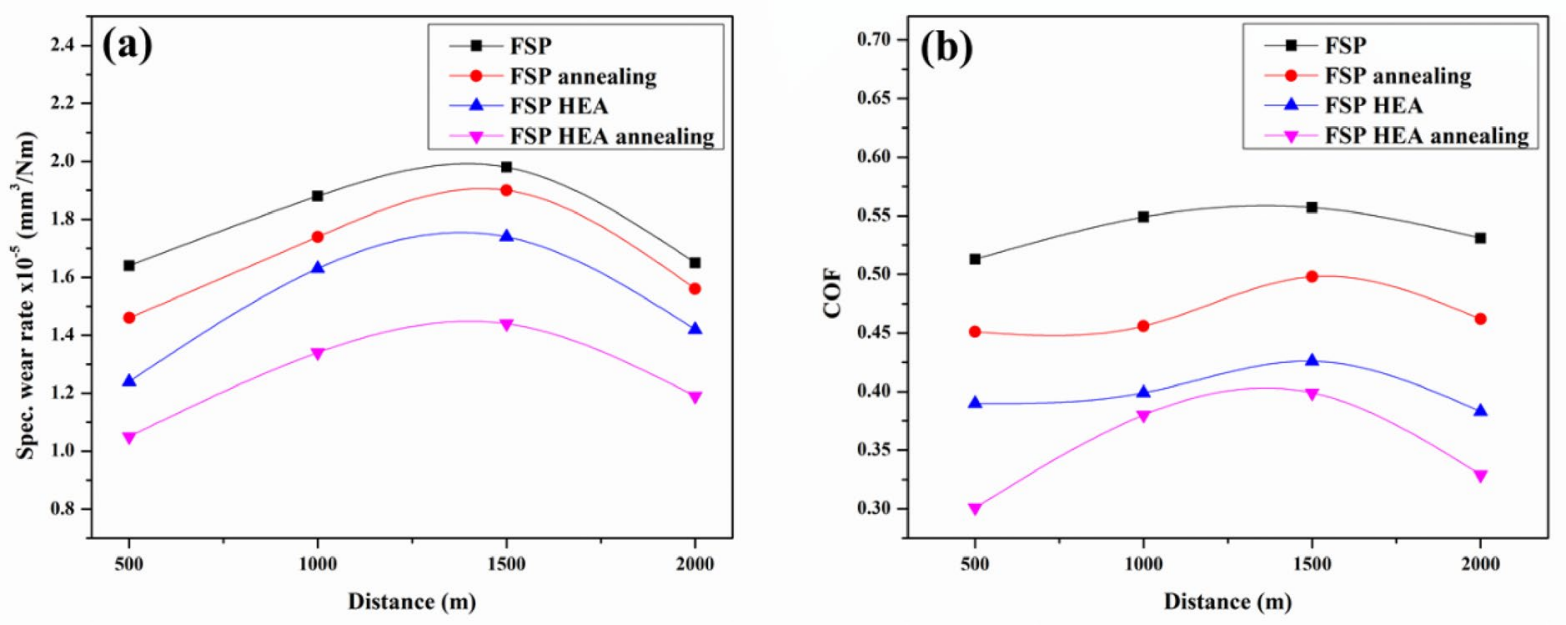
Effect of sliding distance on (**a**) SWR (**b**) COF.

The effect of sliding distance on SWR was analysed for a sliding distance range between 500 to 2000 m with a constant load of 20N and sliding velocity of 1.5 m/s and the corresponding wear trend is depicted in Fig. 9a. It is evident that the SWR of PHM and AHM is much lower than PBM and ABM. The AHM revealed a 15.3% and 35.9% decrement in SWR to the PHM and PBM. The processed sample with and without HEA shows gradually increased material loss initially till 1500 m and decreases further. This is attributed to the formation of a Mechanically Mixed Layer (MML) and surface delamination. The MML formation on the surface aids in improved wear resistance. The MML formed will behave like a defensive layer over the processed surface and subsequent wear will occur on the newly formed layer protecting the processed surface. The wear of the later stages will be transformed into the MML formed over the processed surface. Thus, the wear resistance will improve upon the MML formation. The MML will have high dislocation density which improves the wear resistance. When subjected to continuous sliding contact, the repeated sliding and contact causes plastic deformation resulting in a smooth and hard surface. As the materials become hard, the SWR decreases and results in decreased SWR with increased sliding distance^54^. Also, the addition of HEA helps in cursory deformation thereby decreasing the SWR^55^. The addition of Ti and Cu to the HEA reinforcement advances the formation of oxide layers that protects the surface from wear at an increased distance^56^. The Ti from the HEA reinforcement advances the rapid formation of an oxide layer that is strong enough on the surface to provide better wear resistance. The Fe element forms an oxide layer on maximum condition and acts as the protective layer on the surface in turn decreasing the SWR. Figure 9b illustrates the COF against sliding distance for the processed samples. The COF trend raises from the initial conditions till 1500 m and drops. At a lower sliding distance, the contact pressure between the counter plate and the pin surface is less decreasing the friction between them thereby possessing lower COF. The delaminated surface also acts as a barrier to wear on the surface of the pin. The stress relieved grains after annealing and the reinforced HEA particles offer better resistance to wear and are evident from the COF trend. The MML layer and the hard surface after continuous sliding aid in the decrement of COF at higher distances.

The SWR and the COF values were better for AHM in every wear variable like load, sliding velocity and sliding distance. The wear resistance of AHM is exceptionally better than the processed base sample. The reinforced HEA particles are directed towards this excellent wear resistance of the base metal. The attained outcomes adduce that the CoCrCuFeTi HEA reinforced through FSP offers an improvement in wear resistance and extends its usage in applications.

### Worn surface morphology

The analysis of the worn surface morphology of the ABM and AHM was conducted subsequently since annealing provided the best results and the outcomes are discussed in the following section. The mechanisms associated with the wear behaviour were validated with the worn surface morphology.

**Figure 10:**
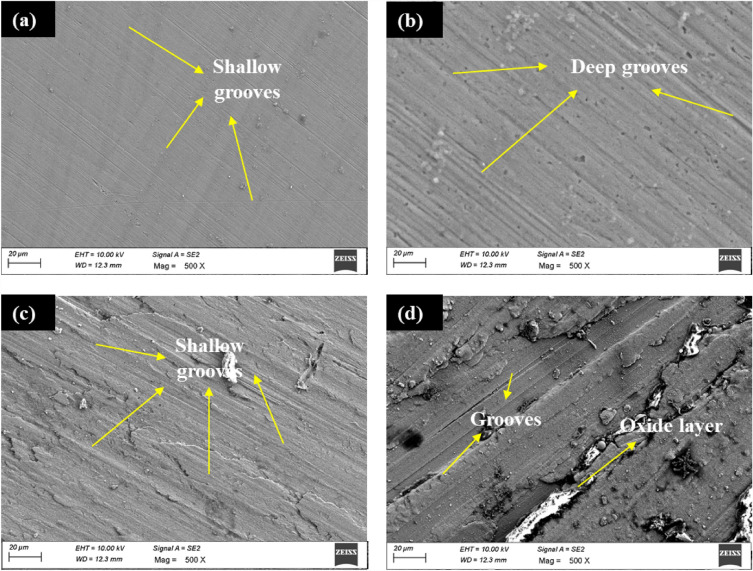
Worn surface of (**a**) ABM at 10N (**b**) ABM at 40N (**c**) AHM at 10N (**d**) AHM at 40N.

The worn surface of ABM and AHM at extreme load conditions is shown in Fig. 10. Figure 10a presents the worn surface of the ABM at minimum load 10N. The abrasive wear mechanism tends to form grooves on the wear surface parallel to the direction of sliding. The SEM confirms the presence of grooves on the surface. With maximum load 40N, the surface of the sample initiated deep groove formation due to the increased contact pressure at the surface (Fig. 10b). The grooves are formed as a result of the micro-ploughing effect between the pin surface and the wear track. However, in the AHM, the surface formed soft grooves at minimum load condition (Fig. 10c). The dislocation density induced by the addition of HEA particles provokes plastic deformation on the surface resulting in the formation of soft grooves^57^. The presence of Cu in reinforced HEA improves the plasticity and tends to make the groove formed softer^58^. The groove became deep at increased load condition (Fig. 10d) caused by the material loss in the form of debris due to high contact pressure. This material loss is the reason for the soft pin surface constantly ploughing through its hard counterpart^59^. The Ti present in the HEA reinforcement has the ability to decrease the micro ploughing effect. A related study has reported that the inclusion of Ti has improved the wear resistance by reducing the micro ploughing effect^56^. The grooves formed on the surface are also shallower compared to the base sample because of the reduced ploughing effect. The improved wear resistance is validated by Archard’s law which correlates the hardness and SWR with maximum hardness offering maximum wear resistance^59,60^. Moreover, the BCC nature of the reinforced HEA and the processed surface aids in improving the wear resistance.

**Figure 11:**
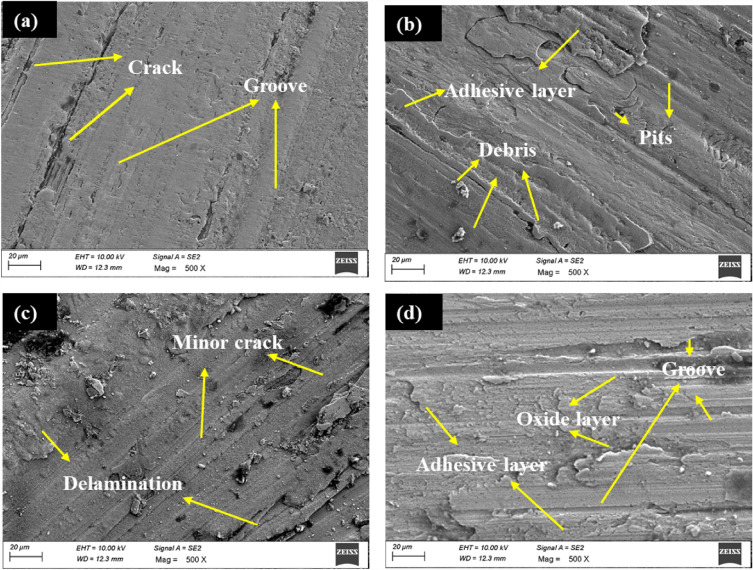
Worn surface of (**a**) ABM at 0.5 m/s (**b**) ABM at 3.5 m/s (**c**) AHM at 0.5 m/s (**d**) AHM at 3.5 m/s.

The worn morphology of ABM and AHM at extreme sliding velocities is shown in Fig. 11. The worn surface of ABM at a minimum sliding velocity of 0.5 m/s is illustrated in Fig. 11a. The worn surface exhibits micro cracks and grooves. The stress developed at the initial condition of sliding velocity induced minor cracks on the surface. At a maximum sliding velocity of 3.5 m/s, the debris from the contact adhered to the surface and the microcracks got propagated forming pits (Fig. 11b). It is also noticed that the wear debris has adhered to the worn surface at high sliding velocity. The worn surface of AHM at a minimum sliding velocity of 0.5 m/s showcased minor cracks and delamination (Fig. 11c). This is due to the rubbing effect of the pin on the hard counterpart. With the maximum sliding velocity at 3.5 m/s, the surface exhibited deep grooves and wedges and some minor oxide layer (Fig. 11d). The increased contact pressure at higher sliding velocity generates a slightly high temperature high enough to form a minor oxide layer. The SWR, compared to the minimum condition, is relatively high but dropped in the maximum condition compared to the intermittent conditions. This is attributed to the formation of a minor oxide layer providing a barrier against wear. The adhesive mechanism drives the formation of an adhered layer of HEA reinforcement and the debris from the counter plate and forms a ridge-like surface. The presence of Cu content in the HEA improves the wear resistance through its self-lubricating property which is reported in a similar study^61^. Also, the better plasticity provided by the presence of Cu led to the groove formation being relatively shallower as opposed to the deep grooves observed in similar studies^62,63^. The presence of Cu and Fe in the HEA form a continuous and smooth oxide glaze layer accompanied by a few delamination evincing the influence of the adhesive wear mechanism^52,64^.

**Figure 12:**
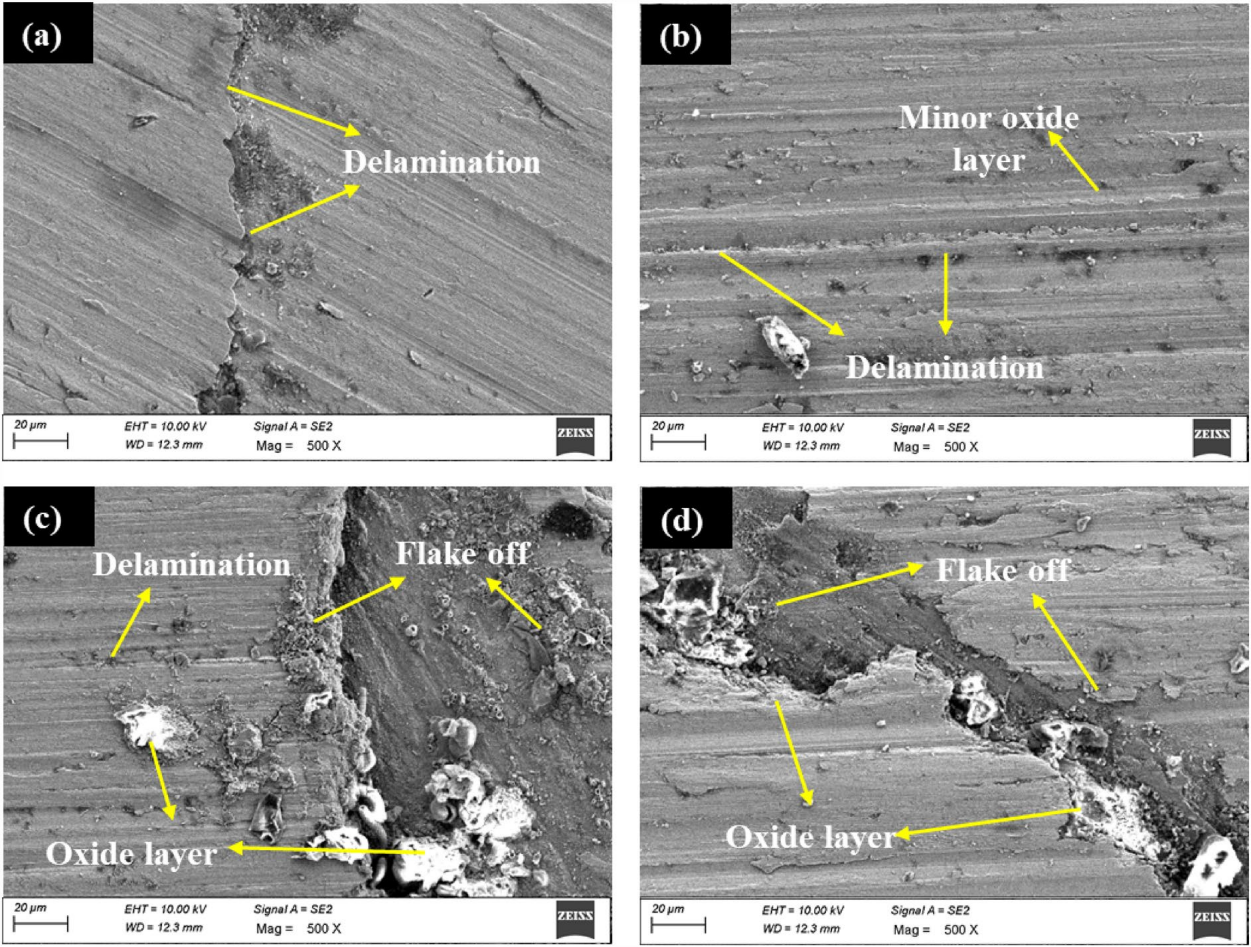
Worn surface of (**a**) ABM at 500 m (**b**) ABM at 2000 m (**c**) AHM at 500 m (**d**) AHM at 2000 m.

The worn morphology of ABM and AHM at extreme sliding distances is depicted in Fig. 12. Figure 12a uncovers the worn surface of ABM at a minimum sliding distance of 500 m, where delamination and a few voids and cracks are observed. The hard surface of the counter plate drives the surface layer to delaminate and initiate the material to flake off from the surface. At a maximum sliding distance of 2000 m, the surface exhibited delamination and grooves with oxide layers (Fig. 12b). The formation of the oxide layer and the hardened surface at increased sliding distance dropped the SWR more than the SWR at the intermittent sliding distances. The morphology of the worn surface of AHM at a minimum sliding distance of 500 m is depicted in Fig. 12c. The surface delamination and the material flake off are noticed from the morphology due to material loss caused by high-stress propagation. The heat generated made the surface to become soft and the high stress generated forced the top layer to initiate peel-off from the surface. Yet, the HEA reinforcement provided enough hardness so that the flake-off was observed to be minimum. Figure 12d depicts the worn surface of the processed sample with HEA at a maximum sliding distance of 2000 m and the formation of oxide layers and smoothened pits are observed. The MML formed defends the wear on the processed surface thereby reducing the SWR as compared to the intermittent sliding distances. The HEA advances the formation of oxide layers and being continuously slid for a prolonged time, the pits and grooves became smooth. The Ti and Fe elements are strong oxygen reactive elements and the presence of those elements in the reinforced HEA advances the oxide layer formation more rapidly^65^. Lower high-temperature softening rates of Ti and Cu in the HEA sustain the hardness and improves the wear resistance. The hard counterpart leads to smoothening of the surface and intense plastic deformation and is reflected by a related study^50^.

## Conclusion

In the current study, FSP was carried out on the SS304 steel by adding CoCrCuFeTi HEA as a reinforcement and the samples were annealed at 650 °C. The outcomes were compared with the processed base samples and samples before annealing. The processed samples were consequently put to microstructural characterisation, microhardness test and tribological analysis.

- The microstructural analysis of the CoCrCuFeTi HEA revealed irregular fragment particles with 17 µm particle size exhibiting the BCC phase and the EDS mapping confirmed the existence of the HEA elements combination.
- The microstructural characterisation of the AHM showed excellent refinement in grains with a homogeneous distribution of HEA particles. The refined grains ranged in nano and sub-micron levels promoting the property enhancements.
- The Vickers microhardness of PHM and AHM was better than the other processed samples. The attained refinement in grains along with the reinforced HEA is credited for this improvement in microhardness.
- The wear test was carried out by varying load from 10 to 40N, sliding velocity from 0.5 m/s to 3.5 m/s and sliding distance from 500 to 2000 m. The wear resistance of AHM was improved by 29.8%, 57.4% and 58.49% at minimum load, velocity and distance than PBM.
- The worn surface was analysed by SEM and the wear mechanisms associated were revealed to be plastic delamination, abrasive wear, adhesive wear and oxide layer formation. The superior grain refinement achieved alongside the HEA reinforcement and the excellent microhardness reinforces the exceptional wear resistance obtained.

The conducted trials evince that the FSP on SS304 with CoCrCuFeTi HEA offers excellent enhancement in mechanical and tribological properties and can be equipped in suitable applications.

## Data Availability

The datasets used and analyzed during the current study available from the corresponding author on reasonable request.
